# A Built-In Strategy to Mitigate Transgene Spreading from Genetically Modified Corn

**DOI:** 10.1371/journal.pone.0081645

**Published:** 2013-12-06

**Authors:** Jing Li, Hui Yu, Fengzhen Zhang, Chaoyang Lin, Jianhua Gao, Jun Fang, Xiahui Ding, Zhicheng Shen, Xiaoli Xu

**Affiliations:** State Key Laboratory of Rice Biology, Institute of Insect Sciences, School of Agriculture and Biotechnology, Zhejiang University, Hangzhou, China; TGen, United States of America

## Abstract

Transgene spreading is a major concern in cultivating genetically modified (GM) corn. Cross-pollination may cause the spread of transgenes from GM cornfields to conventional fields. Occasionally, seed lot contamination, volunteers, mixing during sowing, harvest, and trade can also lead to transgene escape. Obviously, new biological confinement technologies are highly desired to mitigate transgene spreading in addition to physical separation and isolation methods. In this study, we report the development of a built-in containment method to mitigate transgene spreading in corn. In this method, an RNAi cassette for suppressing the expression of the nicosulfuron detoxifying enzyme *CYP81A9* and an expression cassette for the glyphosate tolerant *5-enolpyruvylshikimate-3-phosphate synthase (EPSPS)* gene *G10* were constructed and transformed into corn via *Agrobacterium-*mediated transformation. The GM corn plants that were generated were found to be sensitive to nicosulfuron but resistant to glyphosate, which is exactly the opposite of conventional corn. Field tests demonstrated that GM corn plants with silenced *CYP81A9* could be killed by applying nicosulfuron at 40 g/ha, which is the recommended dose for weed control in cornfields. This study suggests that this built-in containment method for controlling the spread of corn transgenes is effective and easy to implement.

## Introduction

Corn (*Zea mays.* L) is one of the three most widely grown crops worldwide along with wheat and rice [1]. Genetically modified (GM) corn has been increasing in planting acreage since it was commercialized in 1996. GM corn was the second largest biotech crop, after GM soybean, and accounted for 35% of the total corn-planting acreage in the world in 2012 [2]. Herbicide tolerance and insect resistance are two primary input traits for GM plants. GM corn has also been used to produce pharmaceutical protein by a technique known as molecular pharming [3].

Despite the great benefit of using transgene technology in corn, there have been considerable concerns over transgene spreading, particularly the potential for transgene contamination, which is caused by pollen-mediated gene flow [4], [5]. Because corn is a wind-pollinated crop, gene flow via pollen commonly occurs. In spite of the strict management policy in transgenic cornfield tests and in commercial planting, GM corn contamination and unintended transgene spreading still occasionally occurred [6]–[8]. The contamination by GM corn in conventional cornfields often occurred due to unintended seed lot contamination, volunteers, mixing at sowing, cross-pollination, harvest, and trade [9]. In some countries, there is a demand for labeling GM corn food and feed if a threshold value is achieved, 0.9% in EU, for example. In some instances, such as seeds mixing during the sowing period and the presence of volunteers, the adventitious rates of GMO in corn could exceed the threshold value [10]. Volunteer corn could be common in temperate areas. During harvesting, some cobs, cob fragments or isolated kernels may remain in the fields. Depending on the climatic conditions and crop management, the seeds on these kernels from the previous year might germinate, and these GM plants might flower together with that season’s plants [11]. Usually, transgenic plants are difficult to detect, let alone to selectively eliminate the transgenic plants from the non-transgenic ones in a large area of field crops. Furthermore, the gene flow between the transgenic crops and their wild relatives is also an important concern when GM corn is grown in its center of domestication, such as Mexico. Because insect resistant and herbicide tolerant genes confer some advantages under the pressure of insect pests and herbicides, these transgenes might accumulate in the wild relatives over time. To minimize transgene spreading and contamination, new biological confinement technologies are highly desirable in addition to physical separation and isolation measures.

To date, several biological confinement strategies have been developed for containing transgene spreading, such as plastid transformation, male sterility, and gene use restriction technologies (GURTs). Some reviews have evaluated these strategies in detail [12]–[15]. Plastid transformation has been successfully developed in many plants, such as tobacco, tomato and oilseed rape [16]. However, cereals are particularly recalcitrant to plastid transformation [17]. In recent years, some progress has been reported in rice [18]; however, plastid transformation in corn remains unfeasible. Cytoplasmic male sterility (CMS) was suggested as a biocontainment strategy of GM corn pollen [19]. A blend of cytoplasmic male-sterile hybrids and unrelated male-fertile hybrids, which were called Plus-Hybrids, could minimize the release of GM pollen and simultaneously increase the yield [20]. Gene use restriction technologies (GURTs) were developed to produce sterilized seeds, which germinate only when they are exposed to a specific activator molecule [21]. However, these GURTs have not been used in the field for major grain crops, such as corn and rice [12]. Most of the biological confinement mechanisms that were described above were far from being used for commercial production [22]. In addition, some of these confinement mechanisms (such as plastid transformation or male sterility) could not prevent gene spreading that resulted from seed dispersal during cultivation, harvest or transportation.

In previous reports, we described a built-in strategy for the containment of transgenic rice [23]–[25]. Such transgenic rice was created to be sensitive to bentazon by suppressing the expression of a bentazon degradation enzyme, which was encoded by a cytochrome P450 gene *CYP81A6* [23]. This decontamination method could be incorporated into the rice weed control process, and thus, this method is a simple, reliable and inexpensive way to selectively kill transgenic plants in non-transgenic fields.

Nicosulfuron, which is a sulfonylurea herbicide that was developed by DuPont, has been successfully used for weed control in corn [26]. Sulfonylurea herbicides control a wide range of annual and perennial grasses and broadleaf weeds. This type of herbicide has low application rates and displays low levels of acute and chronic animal toxicity [27]. Nicosulfuron has the widest corn safety margin and the fewest sensitive varieties [28]. Tolerant corn plants metabolize nicosulfuron by hydroxylation similar to cytochrome P450 [29], [30]. Previously, the cytochrome P450 enzyme, which is responsible for the detoxification of nicosulfuron in corn, was cloned as *nsf1* (*CYP81A9*, GI: 195612396) by Dam et al. [31] and by our group [32].

In this study, we report a method to create glyphosate tolerant GM corn plants that are sensitive to nicosulfuron. This method was achieved by constructing a T-DNA, which consisted of two functional cassettes: one cassette contained the glyphosate tolerant *5-enolpyruvylshikimate-3-phosphate synthase* (*EPSPS*) gene *G10*, and the other was an RNA interference (RNAi) cassette, which suppressed the expression of the nicosulfuron detoxifying enzyme gene *CYP81A9*. We demonstrated that such transgenic corn could be selectively eliminated by spraying nicosulfuron. Based on laboratory and field test results, we concluded that any hybrid progenies that carry these transgenes could be selectively eliminated by nicosulfuron during the regular weed control process in cornfields.

## Results

### Construction of a T-DNA Plasmid for Creating Terminable Transgenic Corn

**Figure 1:**
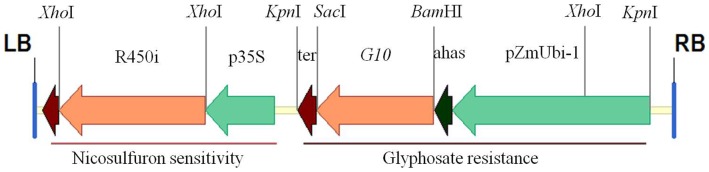
Diagram of T-DNA used for corn transformation. pZmUbi-1, the Z. mays polyubiquitin-1 promoter; ahas, a chloroplast transit signal peptide from a corn acetohydroxyacid synthase gene, which was used to direct the G10 gene to the chloroplast; G10, the corn codon-optimized synthetic glyphosate resistant 5-enolpyruvylshikimate-3-phosphate synthase gene, which was isolated from Deinococcus radiodurans R1;ter, a 3' end terminator fragment from the corn phosphoenolpyruvate carboxylase (PEPC); p35S, cauliflower mosaic virus 35S promoter; 450i, the inverted repeat sequence of a 712 bp fragment of CYP81A9; RB, right border of T-DNA; LB, left border of T-DNA.

A binary T-DNA transformation plasmid was built that was based on pCAMBIA1300. This plasmid contained an RNAi cassette, which targeted the corn cytochrome P450 gene *CYP81A9,* and an expression cassette for the glyphosate tolerant *5-enolpyruvylshikimate-3-phosphate synthase* (*EPSPS*) gene *G10* (GI: 8469109) (Fig. 1). The RNAi cassette included the cauliflower mosaic virus 35S promoter and an inverted repeat sequence of 712 bp of the corn cytochrome P450 gene *CYP81A9*. The *EPSPS* gene *G10* was originally cloned from *Deinococcus radiodurans* R1 and was then further codon-optimized and synthesized for corn expression. The *G10* expression cassette contained the following sequences: the *Z. mays* polyubiquitin-1 promoter (pZmUbi-1), a chloroplast transit signal peptide from the corn *acetohydroxyacid synthase* gene (GB: X63553.1), the codon-optimized synthetic *G10* gene, and a 3' end terminator fragment from corn *phosphoenolpyruvate carboxylase*. The chloroplast transit signal peptide was used to direct the G10 protein to chloroplasts. The RNAi cassette was constructed in tandem to the *G10* gene expression cassette inside the same T-DNA (Fig. 1).

### Corn Transformation and Identification of Transgenic Plants

The corn line “hybrid Hi-II” was used as the recipient of the transgene, and the transformation was conducted using an *Agrobacterium*-mediated approach. A typical protocol for the high-efficiency transformation of maize (*Zea mays* L.), which was mediated by *Agrobacterium tumefaciens*, was used for transformation with only minor modifications [33]. For the transformation, the embryonic tissue from 8–10 day-developing seeds were cultured with the *Agrobacterium tumefaciens* strain LBA4404. To select transgenic events, 2 mM glyphosate was used. In total, 109 independent transgenic events with 375 plants were obtained. All these transgenic plants survived on rooting media, which contained 0.1 mM glyphosate and were all positive in the PCR detection of the *G10* gene, as expected. All T0 transgenic plants were cultivated in a greenhouse and were crossed with the elite corn line Zheng-58 to generate T0×Zheng-58 plants. The T0×Zheng-58 plants were used for further analysis.

### Analysis of the T0×Zheng-58 Transgenic Corn Plants

The hybrid seeds that were harvested from T0 plants were germinated, and seedlings were grown in the greenhouse to test their sensitivity to nicosulfuron and glyphosate. Hybrids between Hi-II and Zheng-58 were used as the control plants. The T0×Zheng-58 plants were divided into two groups for the herbicide spray test. One group of the T0×Zheng-58 plants was sprayed with nicosulfuron at 60 mg/L, and the other group was sprayed with 4 g/L glyphosate at the V4 to V5 stage. Among the 109 transgenic events that were generated, 35 were obviously sensitive to nicosulfuron and exhibited symptoms from varying degrees of injuries, such as stunting and malformation, to death 10 days after nicosulfuron spray. The other events showed no obvious damage that was caused by the application of nicosulfuron.

**Figure 2:**
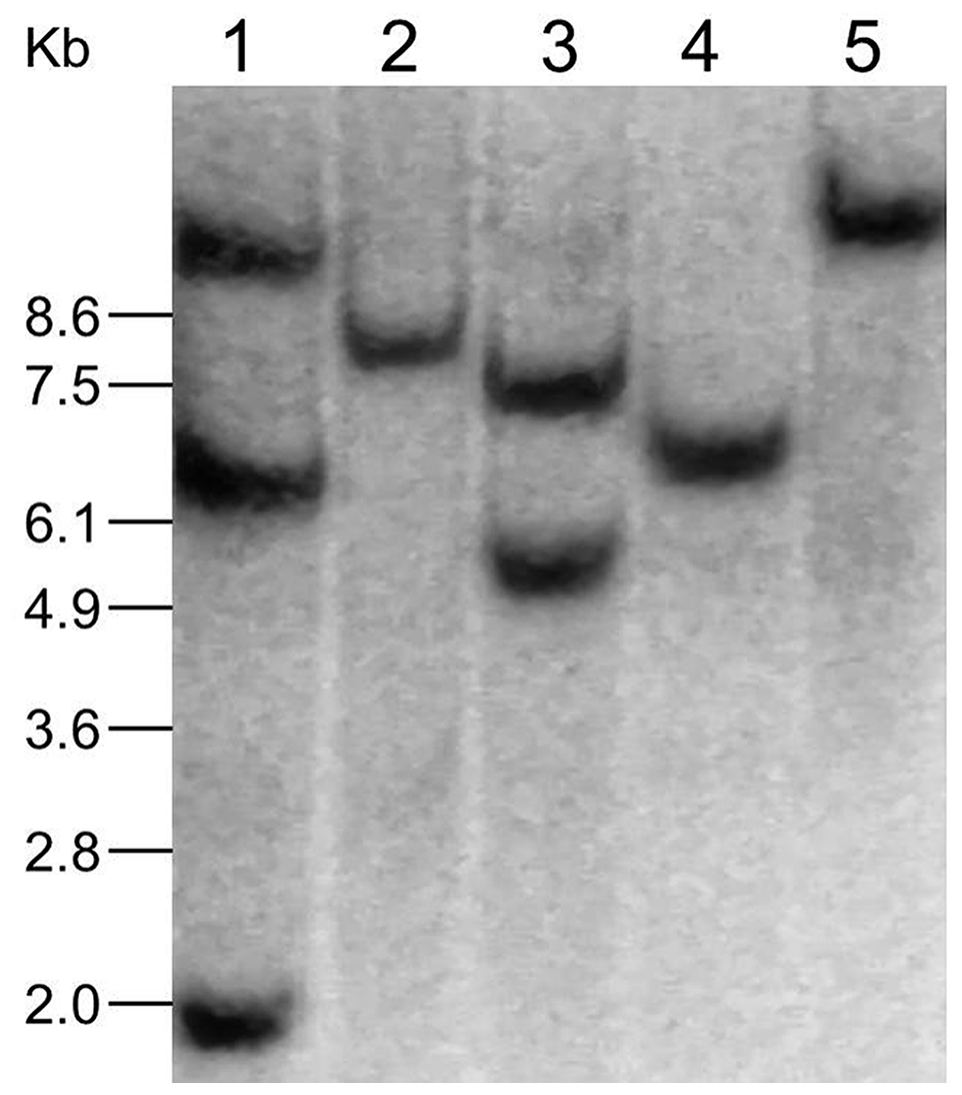
Southern blot analysis of transgenic corn. The genomic DNA of the five independent transgenic events were digested by BamHI and then probed by the synthetic 5-enolpyruvylshikimate-3-phosphate synthase (EPSPS) gene, G10. Lanes 1–5, transgenic events R450-8, R450-42, R450-53, R450-58, and R450-93.

To search for a transgenic event that was sensitive to nicosulfuron and that only had a single copy of the transgene, 5 of the 35 sensitive events were selected for Southern blot analysis. Among the 5 events that were analyzed, events R450-42, R450-58, and R450-93 had a single copy of T-DNA insertions (Fig. 2).

**Figure 3:**
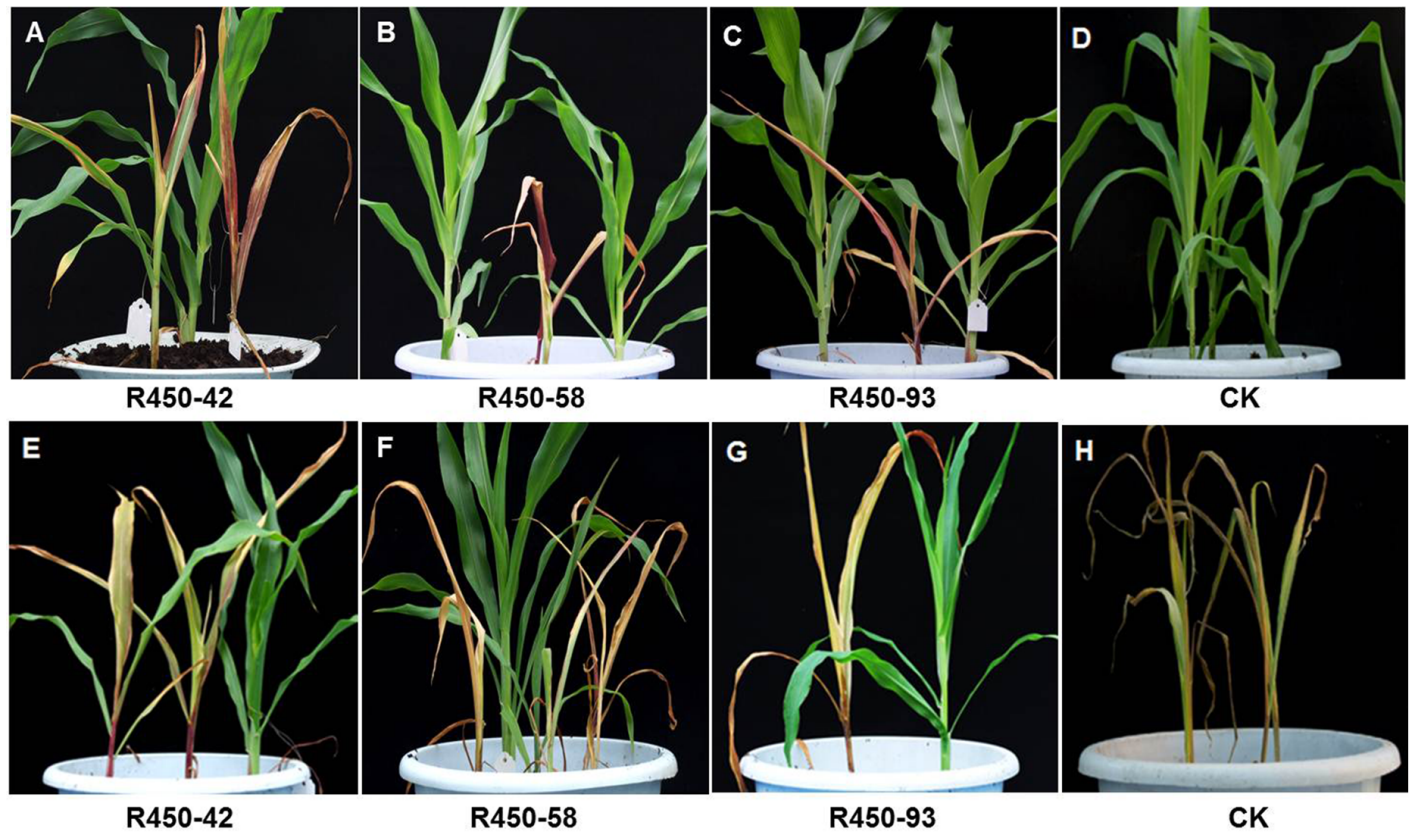
Sensitivity test of transgenic corn plants to nicosulfuron or glyphosate. The T0×Zheng-58 transgenic corn events (R450-42, R450-58 and R450-93) along with non-transgenic ones (CK) were cultured in a greenhouse and sprayed with 60 mg/L nicosulfuron (A–D) or 4 g/L glyphosate (E–H). The photos were taken 10 days after spraying.

Due to segregation, approximately half of the T0×Zheng-58 plants of events R450-42, R450-58 and R450-93 were expected to have the transgenes, which confer sensitivity to nicosulfuron but tolerance to glyphosate. Indeed, as expected, spraying nicosulfuron severely damaged or killed approximately half of the T0×Zheng-58 plants of events R450-42, R450-58 and R450-93 in 10 days (Table 1, Fig. 3A, 3B and 3C); however, spraying nicosulfuron did not incur any visible damage to the non-transgenic control plants (Fig. 3D). In contrast, spraying glyphosate killed all non-transgenic control plants (Fig. 3H), as well as approximately half of the T0×Zheng-58 plants of events R450-42, R450-58 and R450-93 (Table 1, Fig. 3E, 3F and 3G).

**Table 1:** Herbicide resistance of segregating T0×Zheng-58 plants.

Events	Nicosulfuron			Glyphosate		
	Total plants	Surviving plants	Survival (%)	Total plants	Surviving plants	Survival (%)
R450-42	19	10	52.6	16	9	56.3
R450-58	20	9	45.0	18	9	50.0
R450-59	17	8	47.1	15	8	53.3
CK	7	7	100.0	8	0	0.0

The T0×Zheng-58 hybrid plants of R450-42, R450-58 and R450-93 along with non-transgenic ones (CK) were cultured in a greenhouse and sprayed with 60 mg/L nicosulfuron or 4 g/L glyphosate. The data were recorded 10 days after spraying.

**Figure 4:**
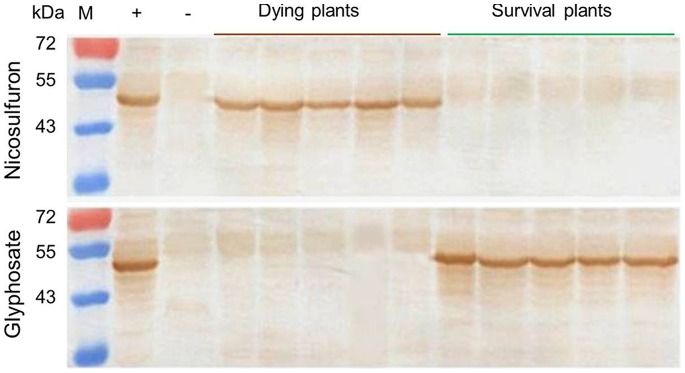
Western blot analysis of G10 (EPSPS) expression in transgenic T0×Zheng-58 plants. The T0×Zheng-58 transgenic corn plants of R450-58 were cultured in a greenhouse and sprayed with 60 mg/L nicosulfuron (upper panel) or 4 g/L glyphosate (lower panel). After 7 days of the treatment, the dying plants and the survival plants were analyzed by Western blotting. −, non-transgenic corn as the negative control; +, G10 protein positive control; M, molecular weight markers.

The plants that were killed by nicosulfuron were expected to contain the transgene, whereas the plants that were killed by glyphosate were segregates without the transgene. To verify this prediction, Western blot analysis was performed on the tested plants of the event R450-58 to detect the G10 protein. The results confirmed that the dying plants, which were affected by nicosulfuron, were the transgenic plants, whereas the survived plants were non-transgenic segregates (Fig. 4, upper panel). The Western analysis further indicated that the plants that were killed by glyphosate were non-transgenic segregates, whereas the survived plants were transgenic (Fig. 4, lower panel). These results clearly demonstrated that the transgenic corn plants that were generated in this study could be selectively eliminated by nicosulfuron.

### RNAi Suppression of the Nicosulfuron Detoxification Gene *CYP81A9*

**Figure 5:**
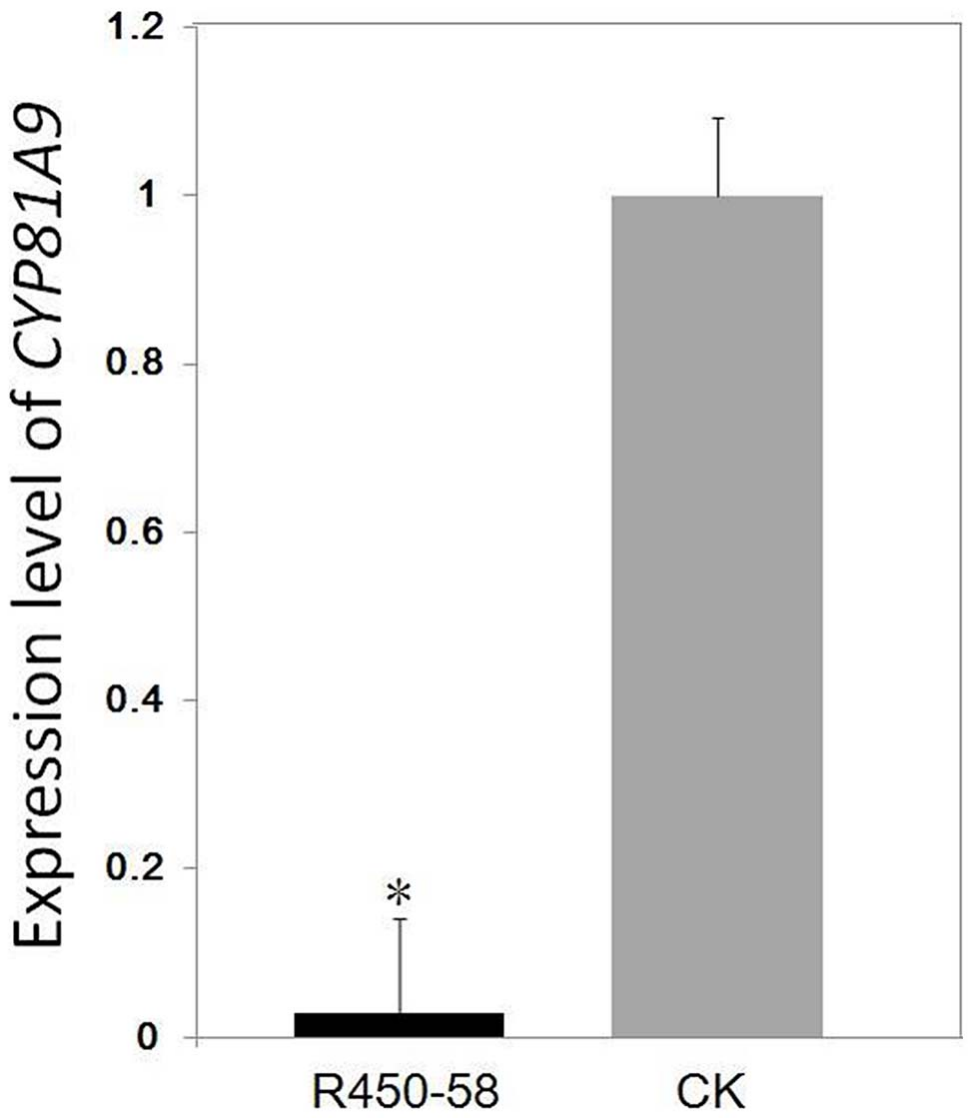
Relative expression of CYP81A9 in transgenic plants (R450-58) and non-transgenic plants (CK). The relative expression levels of CYP81A9 was shown as 2-△△CT, using corn GAPDH as an internal control. The data are expressed as the means of quadruplicates with standard deviations shown by vertical bars. *indicates statistically significant differences between the transgenic corn plants and the non-transgenic control plants under the same conditions (*P<0.05; Student’s t-test).

To analyze the efficiency of the suppression of *CYP81A9* transcripts in transgenic corn plants, qRT-PCR was performed using the *GAPDH* (*glyceraldehyde-3-phosphate dehydrogenase*) gene as an internal control. qRT-PCR analysis revealed that the transcript levels of *CYP81A9* in the event R450-58, which showed high sensitivity to nicosulfuron, decreased by 97.2% compared with its expression levels in non-transgenic control plants (Fig. 5). This result indicated that the expression of the *CYP81A9* gene was drastically suppressed by transforming this RNAi cassette. Similar results were also observed among other independent events that were sensitive to nicosulfuron (data not shown).

### Field Test of the Terminable Transgenic Corn

**Figure 6:**
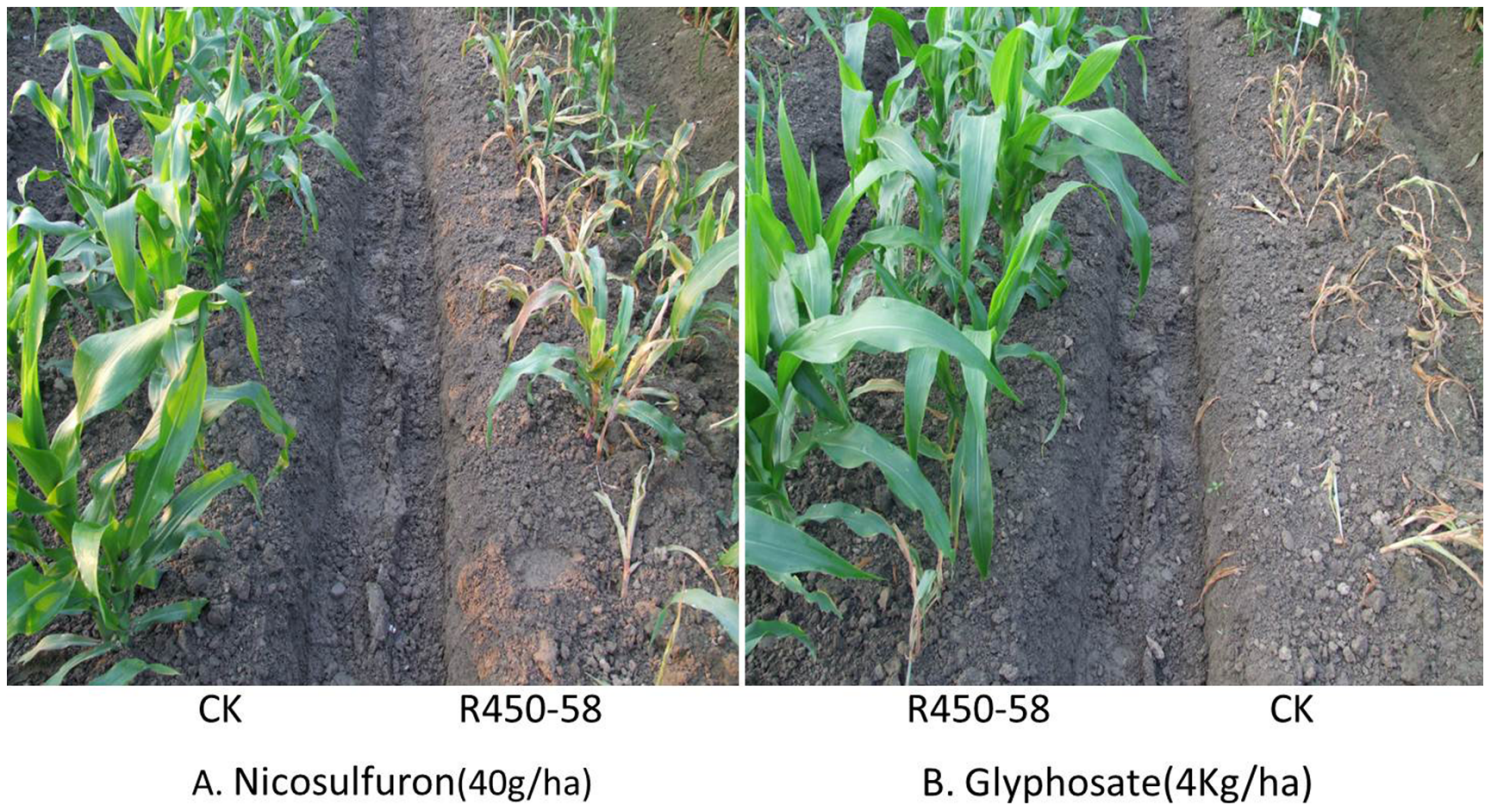
Field trial for T0×Zheng-58 population. The T0×Zheng-58 transgenic plants (R450-58), along with the non-transgenic control plants (CK), were sprayed with 40 g/ha of nicosulfuron (A) or 4 kg/ha of glyphosate (B). The photos were taken 10 days after spraying.

A field test was performed for the further analysis of the sensitivity of T0×Zheng-58 transgenic plants to nicosulfuron and glyphosate. The plants of the event R450-58 were planted in experimental plots in the summer of 2012 in Hangzhou, Zhejiang Province, China. Non-transgenic corn plants that were produced by crossing Hi-II with Zheng-58 were planted along with the transgenic corn as controls. In total, 98 individual corn seedlings of R450-58 were analyzed by PCR before spraying to identify the non-transgenic segregates, which were removed from the field. The segregation ratio was approximately 1∶1 (data not shown), which is consistent with a single T-DNA copy. The planting rows were divided into two parts: one part was sprayed with 40 g/ha nicosulfuron, whereas the other part was sprayed with 4 kg/ha glyphosate at the V5 stage. These application rates were typically used to control weeds under field conditions. Sensitivity was scored 10 days and 20 days after herbicide treatments. All of the transgenic plants were sensitive to nicosulfuron but were resistant to glyphosate. In contrast, the non-transformed control plants were resistant to nicosulfuron but were sensitive to glyphosate (Fig. 6).

Furthermore, there were no significant morphological differences between the transgenic and non-transgenic plants in the field test. The agronomic traits of plant height, ears length, number of kernels per ear and weight per 1000 kernels were measured for the transgenic event R450-58 and the control plants. There were no significant differences (P>0.05) between the transgenic and non-transgenic plants in all these parameters, which were analyzed by Student’s *t*-test using the DPS statistical software (Table 2). This result suggested that the suppression of *CYP81A9* expression did not have statistically significant side effects on the agronomic performance of the transgenic corn in the field.

**Table 2:** Comparison of agronomic traits between the transgenic corn plants (R450-58) and its non-transgenic control plants (CK) in the field trial.

	CK	R450-58	P-value
Plant height (cm)	175.95±1.93	177.90±1.35	0.0707
Ear length (cm)	16.93±0.28	16.36±0.57	0.0521
Kernels per ear	464.33±5.39	454.67±9.48	0.0551
Weight per 1000 kernels (g)	211.62±1.72	213.27±0.95	0.0662

At harvest time, the plant height, ear length and kernels per ear of mature ears were recorded. Mature ears were dried and weighed to determine the weight per 1000 kernels. The values are the means ± SD (n = 6).

P-values indicate statistically insignificant differences between transgenic plants (R450-58) and the non-transgenic control plants (CK) under the same conditions at the 0.05 level, using Student’s *t*-test.

## Discussion

Previously, we reported a built-in strategy for the containment of transgenes in rice [23]. This method was found to be effective in mitigating the spread of transgenes when this method was used in the development of insect-resistant and herbicide-tolerant GM rice [25].

In the study, we demonstrated that the transgenic corn plants that contained the silenced nicosulfuron-detoxifying gene *CYP81A9* were highly sensitive to nicosulfuron and could be selectively killed by spraying nicosulfuron at a regular application dosage. Because nicosulfuron is widely used for weed control in conventional cornfields, any transgenic corn plants that are developed by this method can be selectively decontaminated without extra effort or cost. Therefore, this transgene spreading control method is simple, preventive and could easily be incorporated into the regular weed control process.

In addition to its application in regular transgenic corn, such as transgenic insect-resistant corn, this built-in containment strategy is especially useful for the development of transgenic plants as bioreactors [23], [24]. Corn has many advantages for the large-scale production of recombinant pharmaceutical proteins, which makes corn the widest used cereal for molecular pharming [3]. However, the risk of transgene spreading is relatively high because corn is a wind-pollinated plant. Due to the nature of the pharmaceutical or industrial proteins, it is particularly important to develop a reliable method for the containment of such transgenes. The method that is described in this report could be an ideal technology for minimizing the risk of contamination of such transgenic corn into food and feed supplies.

## Materials and Methods

### Construction of Binary Vector for Corn Transformation

Genomic DNA was extracted from young leaves of corn according to a standard CTAB method [34]. The 712 bp fragment of *CYP81A9* (GI: 195612396) DNA was obtained by PCR using a forward primer 450F (5′-CTCGAGTTCTCCATGCGCCTGGGGACC-3′, with the *Xho*I recognition site underlined) and a reverse primer 450R1 (5′-AGATCTCAGTGATCACAGTGTCAGTGTAGAC-3′, with the *Bgl*II recognition site underlined). This fragment represents the 223 to 934 bp of the genomic DNA from the initial codon. The other 923 bp fragment of *CYP81A9* DNA was amplified by PCR using the primer 450F and the reverse primer 450R2 (5′-AGATCTACAGACTATGTCAACATAAAGCAC-3′, with the *Bgl*II recognition site underlined). This fragment represents the 223 to 1145 bp of the genomic DNA from the initial codon. Both PCR products were separately cloned into the pMD-T vector (Shanghai Sangon, China) and sequenced. Plasmids were digested with *Xho*I and *Bgl*II to obtain the inserts. A three-way ligation was performed to clone these two fragments into pCAMBIA1300, which was predigested with *Xho*I and dephosphorylated by CIAP. The resulting plasmid, which contained a 712 bp inverted repeat sequence of *CYP81A9* for RNA interference, was designated as p1300-450i. Essentially, the *hpt*II gene (hygromycin resistance) in the pCAMBIA1300 vector was replaced with the 450i gene, which was driven by the CaMV 35S promoter for nicosulfuron sensitivity.

The *Z. mays* polyubiquitin-1 promoter (pZmUbi-1) DNA fragment, including the chloroplast transit signal peptide from the *acetohydroxyacid synthase* of *Z. mays*, was generated by digesting a pMD-T plasmid, which contained the promoter with *Kpn*I and *Bam*HI. The corn codon-optimized synthetic glyphosate resistant *5-enolpyruvylshikimate-3-phosphate synthase* (*EPSPS*) gene *G10* (GI: 8469109), including a 3′ end terminator fragment from the corn *phosphoenolpyruvate carboxylase* (PEPC), was synthesized by Shanghai Sangon Limited, China. To facilitate the cloning, the restriction sites of *Bam*HI and *Kpn*I were introduced into the 5′ and 3′ ends of the *G10* gene, respectively. By a three-way ligation, the restriction fragments of the ZmUbi-1 promoter that was fused with the signal peptide and the *G10* gene were inserted into the plasmid p1300-450i, which was predigested with *Kpn*I and dephosphorylated by CIAP. Finally, the direction of the inserts was identified by *Xho*I. Thus, the RNA interference cassette was constructed in tandem with the glyphosate resistance cassette inside the T-DNA. The final plasmid, p1300-450i-G10, with the two expression cassettes in same orientation, was used for corn transformation.

### *Agrobacterium*-mediated Corn Transformation

The plasmid p1300-450i-G10 was transformed into the *Agrobacterium tumefaciens* strain LBA4404 by electroporation using an Electroporator 2510 (Eppendorf) following the manufacturer’s instructions. The hybrid Hi-II corn (*Z. mays* L.) was transformed using a standard *Agrobacterium*-mediated transformation method, which was described previously [33], except that the final concentration of 2 mM glyphosate (Sigma) was used as the selection agent during callus culture and that 0.1 mM glyphosate was used during rooting stage. The independently transformed events were propagated in sterile culture and then planted in soil in the greenhouse for artificial pollinations. T0 plants were crossed with the elite event Zheng-58 to obtain the T0×Zheng-58 seeds.

### Greenhouse Test

The resulting T0×Zheng-58 plants from T0 plants, which were crossed with Zheng-58 and seeds were planted in the greenhouse. Hybrid plants from Hi-II that were crossed with Zheng-58 were used as controls. The amount of the plants that were evaluated for the herbicide resistance was unequal according to the total number of seeds, which were obtained from the events. Briefly, 3–4 plants were planted in one pot (some seeds did not germinate). Plants from an event were planted in 2, 4, 8 or 10 pots for the herbicide spray test. Each of the transgenic events was divided into two groups for the herbicide spray test. One group of the T0×Zheng-58 plants was sprayed with nicosulfuron at 60 mg/L, whereas the other group of the T0×Zheng-58 plants was sprayed with 4 g/L glyphosate at the V4 to V5 leaf stage. A handhold sprayer was used to apply the herbicide formulations. Individual plants were assessed for the nicosulfuron response score 10 days after being sprayed and assigned a visual response score from 1 to 4 (1 = dead plant, 2 = serious damaged plant, 3 = slight damaged plant and 4 = no effect observed). Nicosulfuron (4% suspending solution, Ishihara Sangyo Kalsha Ltd., Japan) and Roundup (41% isopropylamine salt of glyphosate, Monsanto) were used for the herbicide spraying test.

### Western Blot Analysis

A standard Western blot analysis was performed to detect the expression of G10 in both the survival and the dying transgenic corn plants after being sprayed with 60 mg/L nicosulfuron or 4 g/L glyphosate. The samples from all tested plants were collected 7 days after the treatment. Leaf samples that were collected from transgenic plants as well as from non-transgenic control plants were ground to a powder in liquid nitrogen and then suspended in SDS sample buffer. After lysis and centrifugation, the protein samples were separated by 10% SDS-PAGE and then blotted onto nitrocellulose membranes. The rabbit polyclonal antibodies that were generated against G10 were used as the primary antibodies, and the horseradish peroxidase-conjugated goat anti-rabbit IgG was used as the secondary antibody (Promega) in the analysis.

### Southern Blot Analysis

For Southern blot analysis, approximately 100 µg of genomic DNA was digested with the *Bam*HI restriction enzyme. The digested genomic DNA was size-fractionated on a 0.7% (w/v) agarose gel by electrophoresis, transferred onto a positively charged nylon membrane and cross-linked to the membrane at 121°C for 30 min. The hybridization probes, which were specific to the *G10* gene, were prepared as described in the DIG System Manual (Roche). The DNA template that was used for producing the *G10* probe was amplified by PCR using the primers G10-F (5′-CACCTTCGACGTGATCGTGCATCCA-3′) and G10-R (5′-CGAGG TGAGCGAAGAACTGAGGG TAGGA-3′).

### Total RNA Isolation and cDNA Synthesis

To minimize dehydration- and wounding-induced gene expression, leaf samples were quickly excised, wrapped in aluminum foil, and immediately frozen in liquid nitrogen. Total RNA was extracted from 100 mg of leaves using the TRIzol reagent (Invitrogen). The resulting RNA was treated with RNase-free DNase I (Promega) to remove all genomic DNA residues. The RNA concentration and purity were determined using a NanoDrop ND-2000 spectrophotometer (Thermo Scientific). The quality of RNA samples were also confirmed by electrophoresis in 1% agarose gels. Only intact RNA was used for the following reactions. Finally, 2.5 µg of the DNase-treated RNA samples were reversing transcribed into cDNA with an oligo (dT)_ 18_ primer using a Revert Aid First Strand cDNA Synthesis Kit (Fermentas). The synthesized cDNA products were diluted 1∶10 in nuclease-free water before being used as templates in the qRT-PCR analysis.

### qRT-PCR Analysis

Two-step qRT–PCR was performed using a 2×SYBR Green Master Mix (Applied Biosystems®) on an ABI 7500 Real-time PCR System according to the manufacturer’s instructions. The level of *CYP81A9* transcript was normalized using the internal control, the *GAPDH* (*glyceraldehyde-3-phosphate dehydrogenase*) gene. The primers that were used for qRT-PCR were 5′-GCTGGCGACGAGAGCGAAAGTA-3′ and 5′-ATGGCCCATTCCGTCGTGGT-3′ for *CYP81A9* and were 5′-AGCAGGTCGAGCATCTTCG-3′ and 5′- CTGTAGCCCCACTCGTTGTC-3′ for *GAPDH*. The PCR reaction volume was 20 µl, which contained 5.0 µl 10-fold diluted cDNA template, 100 µM of each primer and 10.0 µl of 2×SYBR Mix. The cycling conditions were composed of 10 minutes of polymerase activation at 95°C, followed by 40 cycles of 95°C for 15 seconds, and 60°C for 1 minute. To verify that only a single product was amplified, a dissociation curve analysis was performed using the ABI Prism Dissociation Curve Analysis software. All samples were run on the same plate to avoid between-run variations. For each qRT-PCR experiment, four technical repetitions were performed, and the mean values were calculated. The relative expression levels were calculated using the comparative 2^−△△CT^ method [35] with *GAPDH* as the internal control.

### Field Trials

The R450-58 plants were planted in the experimental plots in the summer of 2012 in Hangzhou, Zhejiang Province, China. Non-transgenic plants, which were prepared by crossing Hi-II with Zheng-58, were planted along with the transgenic corn as controls. The application rates for nicosulfuron and glyphosate were 40 g/ha and 4 kg/ha, respectively. Testing plots were inspected visually 10 days and 20 days post-herbicide application. The number of dead plants was recorded to determine the segregation ratios. Five major agronomic traits including plant height, ears length, number of kernels per ear and weight per 1000 kernels, were recorded from mature plants. Plant heights were measured from the soil surface to the tip of the tassel of each tested plant. Ears length and kernels number were averaged from 6 randomly selected ears from transgenic and non-transgenic control plants.

### Statistical Analysis

All the data were presented as the mean ± SD (standard deviation). Student’s *t*-test was performed to compare the differences between the transgenic and the non-transgenic corn plants. P<0.001 was considered extremely significant, P>0.05 was considered non-significant. All statistical analyses were performed using the DPS statistical software (Refine Information Tech. Inc., Hangzhou, China).
